# Concomitant tonsillar cyst and papilloma in larynx: A case report

**DOI:** 10.1097/MD.0000000000011280

**Published:** 2018-06-29

**Authors:** Qiang Liu, Hong Huo

**Affiliations:** Department of Otolaryngology, Peking Union Medical College Hospital, Peking Union Medical College and Chinese Academy of Medical Sciences, Beijing, China.

**Keywords:** classification, laryngeal cyst, management, papilloma, tonsillar cyst

## Abstract

**Rationale:**

Laryngeal cysts are rare lesions that may occur at any mucosa-lined location within the larynx. Papillomas are also benign lesions of the larynx.

**Patient concerns:**

We report a 34 year-old-patient with a laryngeal cyst incidentally found during screening endoscopy and presenting as a soft tissue mass on a computerized tomography scan. A papilloma concomitant with the cyst was detected intraoperatively.

**Diagnoses:**

Concomitant tonsillar cyst and papilloma of the larynx.

**Interventions:**

The lesion was completely resected with a bipolar radiofrequency plasma ablation (RFA) device.

**Outcomes:**

Pathologic examination showed a tonsillar cyst and papilloma in the larynx. Six months later, there has been no evidence of recurrence.

**Lessons:**

To our knowledge, this is the first report of a concomitant tonsillar cyst and a papilloma in the larynx. Asymptomatic laryngeal cysts can be detected endoscopically. RFA is safe and effective for endoscopic management.

## 1 Introduction

Laryngeal cysts are rare lesions that may occur at any mucosa-lined location within the larynx. Laryngeal cysts have been reported to account for approximately 4.3% to 6% of all benign laryngeal lesions.^[1]^ Newman et al^[2]^ suggested a histologic classification of laryngeal cysts and first described the tonsillar cyst. Tonsillar cysts are characterized by a squamous epithelial lining with underlying follicular lymphoid tissue, resembling tonsillar crypts. Moreover, tonsillar cysts are always located in the valleculae, epiglottis, and pyriform fossa.^[3]^ Papillomas are common benign tumors of the larynx and pharynx and have been associated with viral infections. Although papillomas are benign, they may recur easily. To date, however, there has been no report in the English language literature of any individual patient with a concomitant tonsillar cyst and papilloma. This report describes a 34-year-old patient with a laryngeal cyst that was incidentally found during screening endoscopy, followed by intraoperative identification of a papilloma concomitant with the cyst. The lesion was resected successfully by simultaneous excision, with the pathology results confirming a tonsillar cyst and papilloma.

## 2 Case report

**Figure 1:**
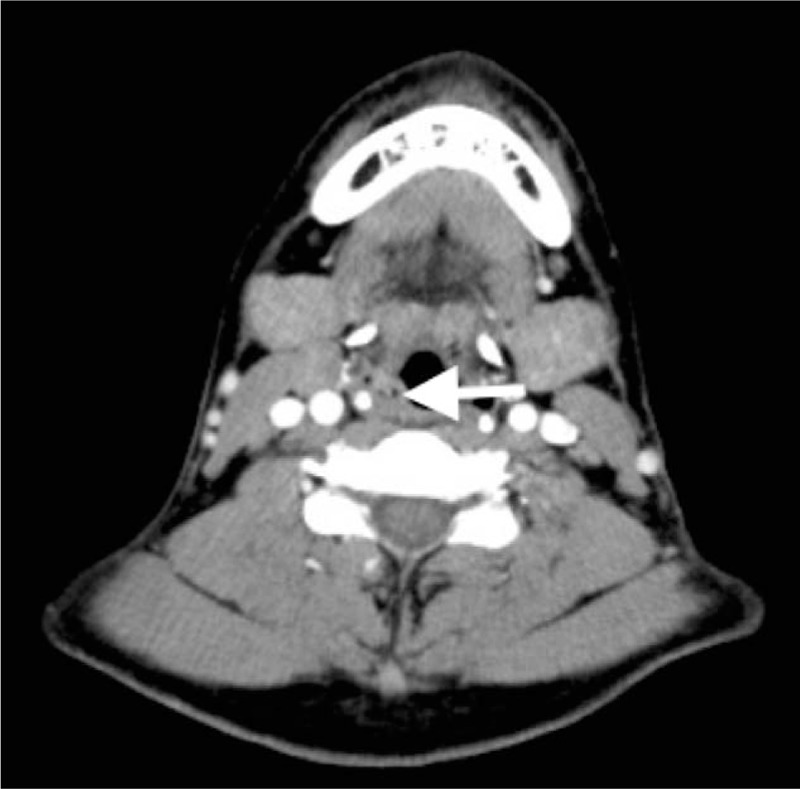
CT scan showing a hypodense mass in larynx (arrowhead).

**Figure 2:**
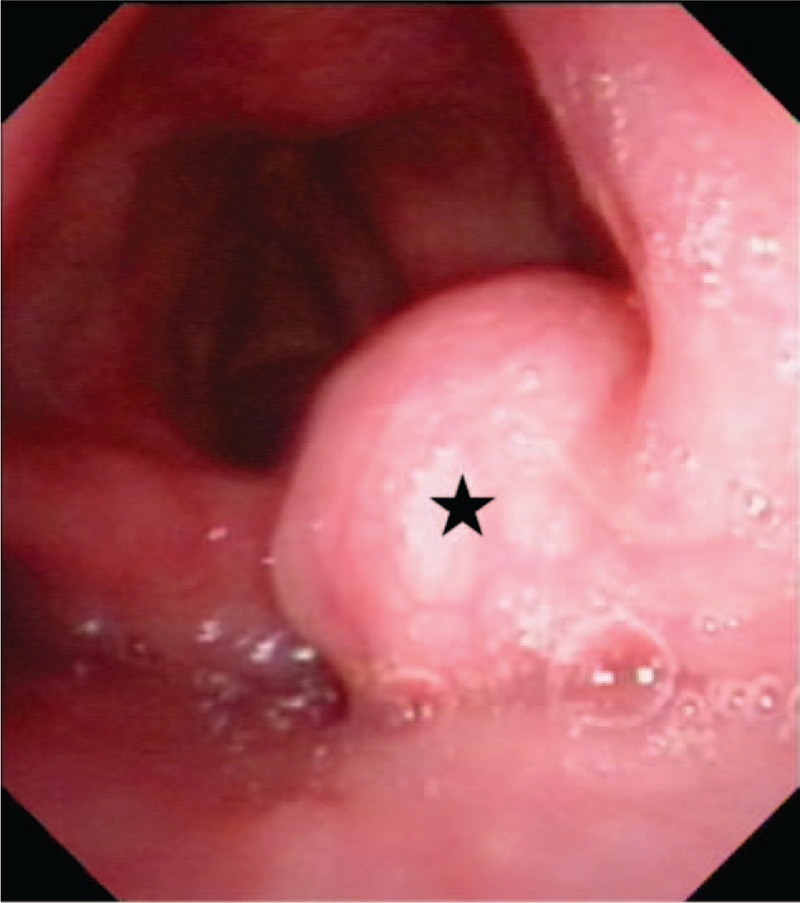
Laryngoscopy showing a round mass on the laryngeal surface and right edge of epiglottis (the black star).

A 34-year-old man was referred with abdominal pain, abdominal distension, and loss of appetite for 6 months. At his first visit to the Department of Gastroenterology, he was evaluated by diagnostic electronic gastroscopy, which detected a mass in his throat. He had no history of dyspnea, dysphagia, odynophagia, foreign body feeling, or hoarseness. He subsequently visited the Department of Otolaryngology for throat discomfort after his last endoscopic examination. Physical examination showed that the patient was healthy-looking in appearance, with other characteristics, including his tonsils, thyroid gland, and lymph nodes, being unremarkable. Computed tomography (CT) imaging showed a mass bulging out of his pharyngolaryngeal cavity (Fig. 1) and flexible fiberoptic laryngoscopy revealed a round laryngeal cyst (Fig. 2).

**Figure 3:**
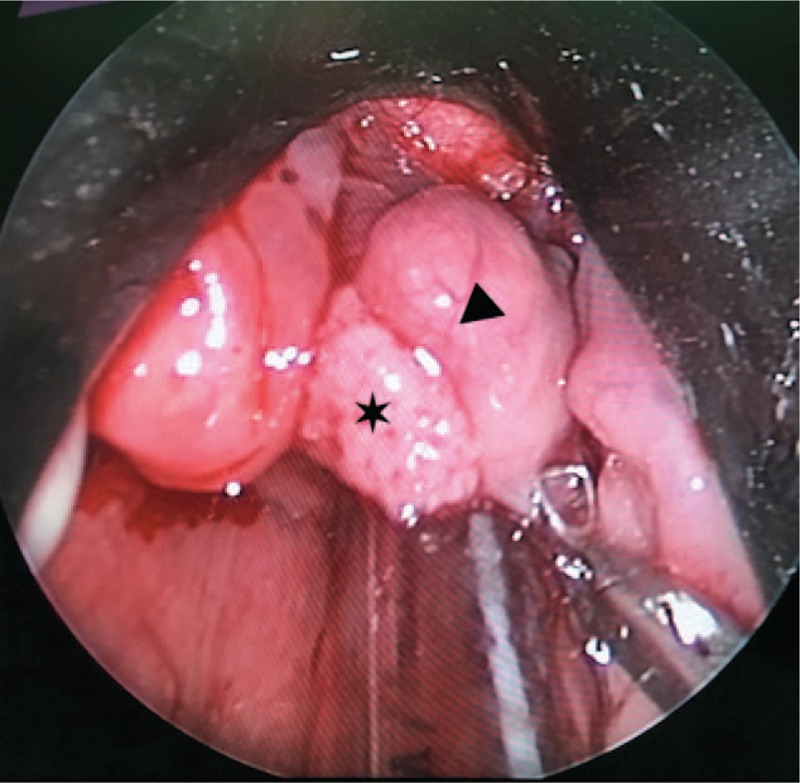
Endoscopy during operation under general anesthesia: the left is papilloma (black star) and the right is tonsillar cyst (black triangle).

**Figure 4:**
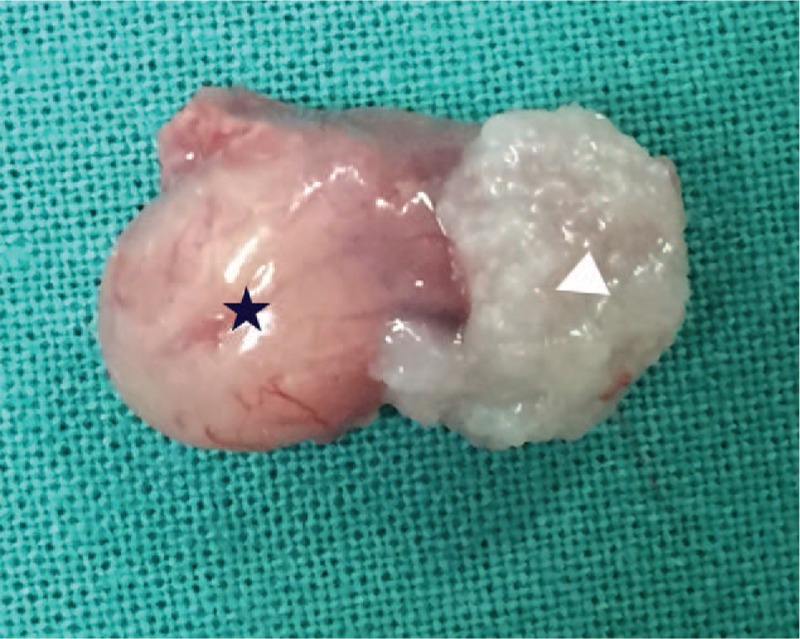
Complete resection of the mass: the left is cyst (black star) and the right is papilloma (white triangle).

**Figure 5:**
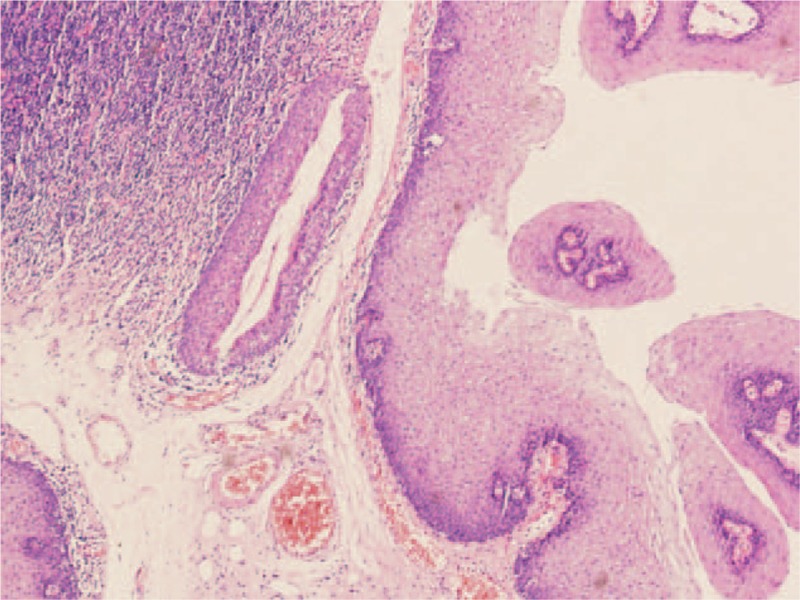
Histopathologic findings. The slide shows the squamous epithelium in papillary arrangement, and under the squamous epithelium there is loose connective tissue with diffuse lymphoid infiltration.

The patient underwent endoscopic excision of the mass. Transoral fiberoptic intubation was performed under general anesthesia. A 2 × 2 cm mass was observed on the laryngeal surface and the right edge of the epiglottis, which appeared to be a cyst. Following suction to remove the cyst at its base, however, another mass was found to emanate from the underside of the cyst. This mass had the appearance of a papilloma, measuring about 1 × 1 cm (Figs. 3 and 4). Hence the patient was diagnosed as having concomitant tonsillar cyst and papilloma of the larynx, and the lesion was completely excised microsurgically using a bipolar radiofrequency plasma ablation (RFA) device. The entire lesion was sent to the pathologist and the pathologic findings showed that the squamous epithelium was in a papillary arrangement, and that under the squamous epithelium there was loose connective tissue with diffuse lymphoid infiltration, which confirmed the diagnosis (Fig. 5). The patient's postoperative course was uneventful, with no evidence of recurrence observed after 6 months. The patient has provided written informed consent for publication of this case report.

## 3 Discussion

Laryngeal cysts are generally benign and have been reported to be more prevalent in women than in men.^[4]^ Congenital laryngeal cysts, first described in 1881, may obstruct airways in neonates.^[5]^ In contrast to laryngoceles, which are air-filled dilatations of laryngeal saccules, laryngeal cysts are fluid-filled dilatations unconnected to the laryngeal ventricle. Although the symptoms of laryngoceles and laryngeal cysts are similar, including hoarseness, cough, stridor, and even dyspnea, depending on lesion size and location, these lesions can be differentiated by CT and magnetic resonance imaging (MRI). CT and MRI are also good for determining the location, size, extent, and anatomical relationships of these lesions.

Laryngeal cysts have been classified into 2 types: ductal and saccular.^[6]^ This classification, however, cannot determine the genesis of the lesions, and is limited to the resulting histologic findings. These cysts have also been classified into 3 types: epithelial, tonsillar, and oncocytic.^[2]^ The lesion in our patient can be classified as tonsillar type. Although tonsillar cysts have been reported to be related to lymphoepithelial cysts of the oral cavity, the origin of tonsillar cysts remains unclear.^[3]^ A third classification system has divided laryngeal cysts into 2 types, with Type 1 cysts confined entirely to the larynx, and Type II cysts extending beyond the larynx.^[7]^Table 1 provides the detailed classifications of laryngeal cysts.

**Table 1:**
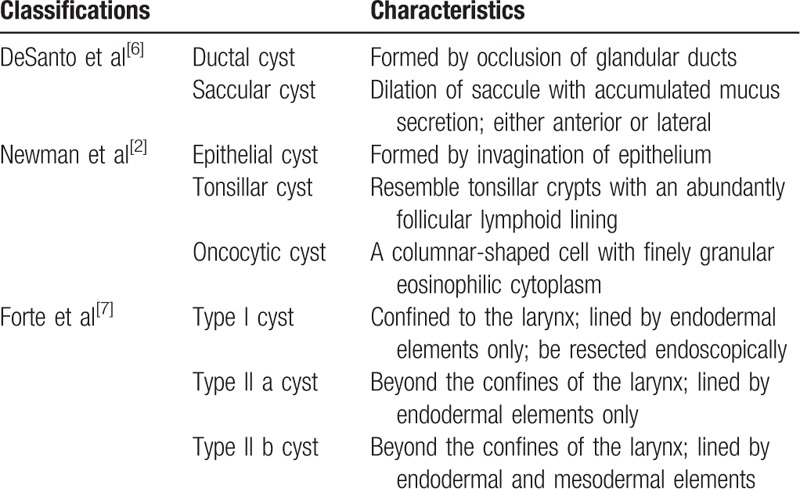
Classifications of laryngeal cysts.

Papillomas are the most frequently occurring benign tumors of the larynx and have been associated with human papilloma virus (HPV). Histologically, papillomas are vascularized, consist of connective tissue covered by stratified squamous epithelium, and project outwardly in finger-like fronds.^[8]^ A patient with a laryngeal saccular cyst caused by recurrent respiratory papillomatosis has been described.^[9]^ Laryngoscopy of this patient showed a large cyst-like mass filling the right ventricle of the larynx, with further examination showing papillomas extending along the false vocal fold and into the ventricle on the side of the lesion, resulting in hoarseness, airway obstruction, and dysphagia. In contrast, our patient presented with a single papilloma originating from papillary hyperplasia of the cyst epithelium, and was asymptomatic.

The main treatment of laryngeal cysts is surgical removal, both by endoscopic management and external approaches. In contrast, laryngoceles may be treated conservatively. Endoscopic procedures are optimal for most endolaryngeal lesions, with the laryngofissure approach often used for lesions that have spread beyond the larynx.^[10]^ It is unclear whether transoral laser marsupialization or complete cyst removal provides better outcomes. Although marsupialization has been reported to be highly successful, the remaining base of the cyst can lead to cyst reaccumulation.^[11]^ Our preference was to completely remove the cyst. Therefore the lesion in our patient was completely excised with RFA, which has been proven safe and effective, with improved surgical precision, minimal bleeding, and better healing.^[12]^

## 4 Conclusion

To our knowledge, this is the first report describing a concomitant tonsillar cyst and papilloma in the larynx. The lesion was completely excised using a bipolar RFA device, which has been shown to be safe and effective. There has been no evidence of recurrence at follow-up.

## Author contributions

**Writing – original draft:** Qiang Liu.

**Writing – review & editing:** Hong Huo.

## References

[1] Lam HC Abdullah VJ Soo G Epiglottic cyst. Otolaryngol Head Neck Surg 2000;122:311. 10652415 10.1016/S0194-5998(00)70264-6

[2] Newman BH Tay JB Laker HI Laryngeal cysts in adults: a clinico-pathologic study of 20 cases. Am J Clin Pathol 1984;81:715–20. 6731351 10.1093/ajcp/81.6.715

[3] Kimura M Nakashima M Nito T Tonsillar cyst of the false vocal cord. Auris Nasus Larynx 2007;34:111–3. 17129695 10.1016/j.anl.2006.09.022

[4] Bhattacharyya N The prevalence of voice problems among adults in the United States. Laryngoscope 2014;124:2359–62. 24782443 10.1002/lary.24740

[5] Abercrombie J Congenital cyst in larynx. Trans Pathol Soc Lond 1881;32:33–4.

[6] DeSanto LW Devine KD Weiland LH Cysts of the larynx—classification. Laryngoscope 1970;80:145–76. 5411821 10.1288/00005537-197001000-00013

[7] Forte V Fuoco G James A A new classification system for congenital laryngeal cysts. Laryngoscope 2004;114:1123–7. 15179225 10.1097/00005537-200406000-00031

[8] Bailey BJ Bailey KH Derkay CS Head and Neck Surgery-Otolaryngology. 2001;Philadelphia: Lippincott Williams & Wilkins, 659–665.

[9] Michael A Saccular cyst caused by recurrent respiratory papillomatosis. Otolaryngol Head Neck Surg 1999;121:668. 10547496 10.1016/S0194-5998(99)70082-3

[10] Xiao Y Wang J Ma JY The clinical characteristics of congenital laryngeal saccular cysts. Acta Otolaryngol 2016;136:168–71. 26507793 10.3109/00016489.2015.1100327

[11] Richard H David G Lott laryngeal cysts in adults: simplifying classification and management. Otolaryngol Head Neck Surg 2017;157:928–39. 28695764 10.1177/0194599817715613

[12] Kumar S Garg S Sahni JK Radiofrequency ablation of laryngeal saccular cyst in infants: a series of six cases. Int J Pediatr Otorhinolaryngol 2012;76:667–9. 22398119 10.1016/j.ijporl.2012.01.039

